# Off-label prescription of psychiatric drugs by non-psychiatrist physicians in three general hospitals in Germany

**DOI:** 10.1186/s12991-018-0176-4

**Published:** 2018-02-08

**Authors:** Caroline Lücke, Jürgen M. Gschossmann, Teja W. Grömer, Sebastian Moeller, Charlotte E. Schneider, Aikaterini Zikidi, Alexandra Philipsen, Helge H. O. Müller

**Affiliations:** 1 Medical Campus University of Oldenburg, School of Medicine and Health Sciences, Psychiatry and Psychotherapy-University Hospital, Karl-Jaspers-Klinik, Hermann-Ehlers-Straße 7, 26160 Bad Zwischenahn, Germany; 2 Department of Internal Medicine, Klinikum Forchheim, Forchheim, Germany; 3 Department of Psychiatry and Psychotherapy, Friedrich-Alexander-University Erlangen-Nuremberg, Erlangen, Germany

**Keywords:** Off-label prescription, Psychotropic medication, Risk profile

## Abstract

**Background:**

Off-label prescribing of psychoactive drugs is a common practice in psychiatry. Here, we sought to investigate the frequency of off-label prescribing in a population of hospitalized patients with a somatic illness who were also suffering from a psychiatric pathology.

**Methods:**

Using a prospective, observational design, we collected data from 982 hospitalized patients with a somatic illness for whom a psychiatric consultation was requested because of the presence of additional psychiatric symptoms. Data were collected at three hospitals in Germany. Demographic and clinical data, including the previous psychoactive medications and an assessment of the suitability of the previous medications, were recorded and analyzed.

**Results:**

Data on the previous psychiatric medications were available for 972 patients. In 16.6% of patients, at least one psychoactive drug had been prescribed off-label, 20.2% had received on-label medication, and 63.2% had not received any psychiatric medication. Among all patients receiving psychiatric medication, 45.1% had received off-label medication. The logistic regression analysis showed a significant influence of age on the likelihood of receiving off-label medication (*p* = 0.018). Benzodiazepines were the most frequent off-label prescription (25.8% of off-label prescriptions), followed by atypical antipsychotics (18.2%) and low-potency antipsychotics (17.2%). Notably, 57.1% of off-label prescriptions were judged to be ‘not indicated’ by experienced psychiatrists.

**Conclusions:**

Our data show a high frequency of the off-label prescription of psychoactive drugs by physicians treating patients with somatic illnesses in general hospitals. Because more than half of these cases were judged to be “not indicated”, these prescriptions indicate a potential risk to patients. Furthermore, the classes of drugs that were most frequently prescribed off-label, benzodiazepines and antipsychotics, both show a substantial risk profile, particularly for elderly patients.

## Background

Off-label prescribing, the prescription of drugs not licensed for the intended use in the country of conduct, is a common phenomenon. The term “off-label” not only refers to the use of a medication outside its licensed indication but also includes the use of dosages above the recommended range, a treatment duration for periods longer than recommended or the use in special patient groups, such as children, the elderly, or patients with contraindications. Moreover, the use of different routes of administration or a change in the formulation, i.e., when a tablet is crushed to be easier to swallow, may result in different pharmacokinetics and constitute an off-label use. Pediatrics is the subspecialty with the largest number of off-label prescriptions due to the special age group of the patients [1, 2], but clear evidence exists for extensive off-label use in psychiatry, as well [3]. According to a cross-sectional questionnaire-based survey from the United Kingdom (UK), 65% of respondents had been prescribed a medication for an off-label indication in the past month [4]. A subsequent UK study analyzing inpatient prescriptions in psychiatric wards reported an off-label prescription rate of 7.5% [5], whereas another study conducted in a German Psychiatric hospital found that 20% of prescriptions were clearly off-label and another 19% of prescriptions were classified as “probably off-label” [6]. However, these studies were conducted more than 15 years ago. More recently, 40% of drugs were prescribed off-label in a prospective study conducted at an Indian outpatient psychiatric department, with 79% of patients receiving at least one off-label drug [7]. Classes of drugs that are commonly prescribed off-label very in psychiatric practice are antipsychotics [5, 8] and benzodiazepines [7, 9], the latter of which is often being prescribed off-label for long-term use.

In some situations, off-label prescribing is a feasible or even necessary practice. Conditions for the use of off-label medications in accordance with professional organizations in most countries include the lack of an equally safe and effective licensed alternative, the existence of sufficient scientific evidence to support the use of the drug for the intended condition, and the presence of fully informed consent from the patient to be treated off-label [10].

However, in many cases, these requirements are not or are only partially fulfilled [10, 11]. Often, the level and quality of the scientific evidence supporting the intended off-label use of a drug is overestimated by physicians [12], and the possible risks of side effects are underestimated [13]. In particular, when drugs are used in off-licensed age groups, such as geriatric patients, the risk of side effects may increase considerably [14]. Although the bias due to the underreporting of adverse events is difficult to estimate, the rate of spontaneous reporting seems lower for unlicensed medicines compared to licensed medicines [13], which may be due to the fear of legal consequences by physicians. Furthermore, patients are often not informed about the unlicensed status of the drug that they receive [15, 16].

In the present analysis, we specifically examined the number of off-label prescriptions of psychotropic drugs for patients who were hospitalized due to a somatic illness and showed additional psychiatric pathology. Psychopharmacological prescriptions were administered to this population prior to specific psychiatric consultations in nearly all cases by the physician treating the somatic illness. Since physicians in a somatic setting typically do not have extensive psychiatric training, the off-label prescription of psychiatric drugs in this setting might constitute a particular risk of the non-optimal or even hazardous treatment of patients.

## Methods

In a prospective, observational design, data from 982 hospitalized patients with a somatic illness were collected and evaluated. Only patients who showed psychiatric symptoms and thus required a psychiatric consultation were included. Patient data were collected from three different hospitals in Germany; 345 cases were from a large university clinic (University Clinic Erlangen) and 636 cases were from two medium-sized general hospitals (545 from Klinikum Forchheim and 91 from Evangelisches Krankenhaus Oldenburg).

Data were collected by the consultation-liaison psychiatrist who conducted the psychiatric consultation and comprised the demographical and clinical psychiatric data, including current psychopathology, previous treatments, psychiatric diagnosis (current and pre-known), and recommended treatment. For patients who previously received a psychiatric treatment, the psychiatrist also evaluated and recorded whether this treatment was medically justified in the given situation. Furthermore, the psychiatric diagnosis suspected by the somatically treating physician prior to the psychiatric consultation was recorded in addition to a judgement by the psychiatrist regarding whether this diagnosis was correct.

All patients received information about the study design from the psychiatrist and provided informed consent to participate. All data were assessed as part of the routine psychiatric consultation, and participating patients did not undergo any additional study-related procedures.

Study results on psychiatric pathology, diagnoses, and further treatment have already been published elsewhere [17]. The current psychoactive medications used by the patients prior to treatment recommendations by the psychiatrist were recorded and classified into groups in the database. Drug prescriptions were then classified as on- or off-label according to the drug’s license in Germany at the time of use.

Data were collected from September 2011 to April 2012 at the University Clinic Erlangen, from March 2014 to September 2015 at Klinikum Forchheim and from January 2016 to September 2017 at Evangelisches Krankenhaus Oldenburg. All psychiatric consultations performed at these clinics during the specified periods were included in the study, unless the patients did not consent to data collection. The physicians treating the somatic illness usually made the decision to request a psychiatric consultation for patients, based on the current psychopathology shown by the patients. Over 90% of consultations were conducted by the same three psychiatrists. The majority of the patients (85.3%) came from the department of internal medicine.

Data were processed and analyzed using SPSS Statistics 23 (IBM, Armonk, NY, USA). Since the design of this analysis was exploratory and based on a large database with several study endpoints, data were primarily analyzed using descriptive statistics. In addition, influences of co-factors such as gender and age were tested using multi- or binomial regression analyses.

This study was approved by the ethics committee of the Friedrich-Alexander-University of Erlangen-Nuremberg.

## Results

Nine hundred and eighty-two patients participated in this study, and information on the previous psychiatric medications was available for 975 patients. Of these patients, 60.3% were female and 39.7% were male. The mean age was 64.3 years.

The rate of off-label prescriptions for psychoactive medications in our patient population was 16.6%. On-label psychiatric medications were prescribed to 20.2% of patients, whereas 63.2% of patients had not received any previous psychiatric medication. When restricting the analysis to patients who had received psychoactive medications, the rate of off-label prescriptions was 45.1%.

A logistic regression analysis was performed to examine the influence of the factors age and gender on the likelihood of off-label prescriptions in patients. The models showed a statistically significant influence of age on the comparison of “on-label use” versus “off-label use” (*p* = 0.018) and the comparison of “no psychiatric medication” versus “off-label use (*p* = 0.047); patients who received off-label prescriptions were slightly older than the other two groups (Table 1). The ratio of male-to-female patients was nearly equal in all groups, and the logistic regression analysis did not show a statistically significant influence of gender (Table 1).

**Table 1:** Demographics and psychiatric medication

	On-label use	Off-label use	No psychiatric medication	Total
Gender				
Male				
Count	76	62	249	387
Percent within psych. medication (%)	38.8	38.3	40.6	39.9
Female				
Count	120	100	364	584
% within psych. mediation (%)	61.2	61.7	59.4	60.1
Mean age (years)	62.9*	67.5*^,+^	64.2^+^	64.3
Total				
Count	196	162	613	971
Percent within psych. medication (%)	100.0	100.0	100.0	100.0

* p = 0.018,^+^ p = 0.047

Among all the previous off-label prescriptions, 57.1% were judged as ‘not indicated’ by the liaison psychiatrist and additional 37.5% were judged as ‘partially indicated’. Only 3.4% of off-label prescriptions were judged to be ‘indicated’.

The distribution of drug classes that were prescribed off-label is shown in Table 2. Benzodiazepines were the class of drugs that were most frequently prescribed off-label, with 25.8% of off-label prescriptions, followed by atypical antipsychotics (18.2%) and low-potency antipsychotics (17.2%). Selective serotonin reuptake inhibitors constituted 12.0% of off-label prescriptions. Taking together all antipsychotics of any generation and potency, antipsychotics were the most frequently prescribed off-label drug class, with 38.3% of off-label prescriptions.

**Table 2:** Off-label prescriptions of psychiatric drugs

Class of drug	Responses	
	*N*	Percent (%)
Low-potency antipsychotics	36	17.2
Atypical antipsychotics	38	18.2
Typical antipsychotics	6	2.9
Benzodiazepines	54	25.8
Other hypnotics	6	2.9
SSRI	25	12.0
Mirtazapine	9	4.3
Tricyclic antidepressants	7	3.3
Lithium	2	1.0
Pregabalin	6	2.9
Others	20	9.6
Total	209	100.0

Psychiatric disorders with high rates of off-label prescriptions were somatoform disorders (28.0%), organic mental disorders (21.9%), and anxiety disorders (22.1%). Sleep disorders and drug-related disorders had high rates of off-label prescriptions as well, but too few patients were included in these categories to provide a valid result. In the single case of a patient with the diagnosis of sleep disorder who had received psychiatric medication, an atypical antipsychotic had been prescribed off-label. Table 3 shows the rates of off-label prescriptions in the different psychiatric diagnoses as determined by the psychiatrist as a result of the consultation.

**Table 3:** Off-label prescriptions in different psychiatric diagnoses

	Psychiatric medication		
	On-label use	Off-label use	No psych. medication
Organic mental disorders			
*N*	37	41	109
%	19.8	21.9	58.3
Alcohol-related disorders			
N	11	17	72
%	11.0	17.0	72.0
Other drugs			
*N*	4	5	14
%	17.4	21.7	60.9
Psychosis			
*N*	13	3	26
%	31.0	7.1	61.9
Affective disorders			
*N*	71	51	223
%	20.6	14.8	64.6
Anxiety disorders			
*N*	16	15	37
%	23.5	22.1	54.4
Reaction to severe stress/adjustment disorder			
*N*	8	14	70
%	8.7	15.2	76.1
Dissociative and conversion disorders			
*N*	0	1	7
%	0.0	12.5	87.5
Somatoform disorders			
*N*	11	14	25
%	22.0	28.0	50.0
Eating disorders			
*N*	1	1	4
%	16.7	16.7	66.7
Sleep disorders			
*N*	0	1	2
%	0.0	33.3	66.7
Other diagnoses			
*N*	1	4	17
%	4.5	18.2	77.3
No psychiatric disorder			
*N*	4	4	40
%	6.4	8.5	85.1

To investigate whether the high percentage of unjustified off-label prescriptions was related to a high amount of incorrect diagnoses, we also analyzed the suspected psychiatric diagnoses made by the non-psychiatrist physicians prior to the psychiatric consultation. Among all cases of off-label prescription for which these data were available (*n* = 104), 48.1% of diagnoses were classified by the psychiatrists as “correct”, 35.6% were “partially correct” (e.g., depressive episode versus adjustment disorder), and 16.3% were “incorrect”.

Furthermore, we performed an exploratory analysis of the distribution of off-label prescribing in the different medical specialties. The vast majority of participants were seen in the internal medicine department, and 74.5% of off-label prescriptions were written by physicians in internal medicine. However, compared to other specialties, internal medicine was slightly below average, with 14.5% of patients receiving off-label medications. The specialty with the highest rate of off-label prescriptions was the emergency department (62.5%), followed by trauma surgery (27.8%), neurology (26.3%), and intensive care (20.0%). Patients from other departments were very rarely prescribed off-label medications; thus, the numbers were too small for interpretation.

## Discussion

To our knowledge, this study represents the first large and systematic analysis that specifically examines the off-label prescribing of psychiatric drugs by non-psychiatrists in a naturalistic clinical hospital environment. We observed a surprisingly high rate of off-label prescriptions in this population, with 16.6% of patients receiving off-label psychoactive medications. Among the patients who received any psychopharmacological medication, 45.1% received at least one off-label drug. Compared to the literature, this rate is clearly higher than the rates of off-label prescriptions reported in British and German psychiatric wards in the previous studies [5, 6]. Several possibilities may explain this discrepancy. The difference may be partially due to a general increase in the number of off-label prescriptions since the early 2000s. Unfortunately, more recent data from Western populations are not available for comparison, which is a surprising finding of the lack of (needed) systematic examinations in the field of in-house off-label prescribing.

Second, differences between the populations studied (psychiatric wards versus medical wards) could account for the discrepancy, since the distribution of psychiatric diagnoses in our population differed from the distribution typically found in psychiatric wards, particularly for organic mental disorders. However, an additional reason for the high rates of off-label prescriptions observed in our population may be because prescribers in our population were mostly physicians from specialties other than psychiatry. These physicians may either have a generally more uncritical view of off-label prescribing or are simply less informed about the licensed indications and the recommended use of the drugs prescribed. If the latter is true, it implies a potential risk for patients, since a responsible off-label prescribing practice requires good knowledge of the scientific evidence supporting the off-label use of the drug in question for a specific condition. Those facts also highlight the need for the systematic education of non-psychiatric prescribers in the field of psychopharmacological treatment options. In some cases, an “unjustified” off-label prescription may have simply been the result of an incorrect psychiatric diagnosis by the prescriber prior to the psychiatric consultation. Our data show that the rate of “incorrect” diagnoses among cases of off-label prescription was 16.3%, whereas a further 35.6% were only “partially correct”. Thus, a prescription may have been on-label based on the physician’s diagnosis at the time of prescription, but off-label based on the correct diagnosis made by the psychiatrist. This effect may have further contributed to the high rate of off-label prescriptions in our patient population. Since the distribution of correct versus incorrect diagnoses was similar in cases of on-label prescription and in cases of no psychiatric medication, we do not believe that this is the primary explanation for our main results regarding off-label prescribing. Nevertheless, the high amount of incorrect or partially correct diagnoses further highlights the necessity of a qualified psychiatric diagnosis prior to prescription of psychopharmacological medication in hospitals.

Our results on the evaluation of previous off-label treatments by specialists support the hypothesis that the frequent off-label prescribing practice in hospitals is very often not in the patients’ interest. More than half of off-label cases were judged to be ‘not indicated’ by psychiatrists, and the off-label prescription was only clearly advisable for 3.4% of patients. These results are consistent with the previous studies, showing that the majority of off-label prescriptions, particularly for psychiatric indications, are not supported by solid scientific evidence [12, 15, 18]. The fact that the justification of off-label prescribed medications was based on the psychiatrists’ assessment and not controlled by standardized rules or a literature search may constitute a certain limitation of our study; however, all psychiatrists performing the consultations were experienced specialists with fundamental scientific knowledge in the area of psychopharmacology.

Consistent with the literature, the classes of drugs that were most frequently prescribed off-label were antipsychotics and benzodiazepines. The off-label prescription of antipsychotics is very common in psychiatry [11, 19–21] and has significantly increased over the years [22, 23]. With some exceptions, atypical antipsychotics are often only licensed for use in patients with schizophrenia and often bipolar disorder, but they are frequently prescribed for other indications as well, particularly anxiety, affective disorders, and organic mental disorders [19, 24, 25], due to their stabilizing effects on agitation. Since the development of atypical antipsychotics with reduced risks of extrapyramidal symptoms (EPS), the reputation of antipsychotics has significantly improved; however, the second-generation antipsychotics may also have significant side effects, particularly regarding metabolic changes. These changes may be negligible in relation to the immediate dangers of acute psychosis or manic phases, but the uncritical use of antipsychotics in other, less threatening conditions may not always be justified. Furthermore, extrapyramidal side effects may still occur with atypical antipsychotics [14], and tardive dyskinesia is of particular concern because of its potential irreversibility. In a 2006 prospective cohort study of patients receiving maintenance antipsychotic treatment, the risk of tardive dyskinesia following treatment with quetiapine was as high as 13% [26]. The study also confirmed previously known risk factors for the development of EPS, including an older age and duration of treatment [26–28]. Other potentially serious risks of antipsychotics are cardiac side effects [29–31] which may be of particular concern in patients with preexisting heart disease. Thus, uncritical off-label use of antipsychotics seems especially problematic in elderly populations, such as our study population [32].

Long-term benzodiazepine use causes tolerance, addiction, and potential abuse; thus, licenses and national guidelines usually recommend the prescription of benzodiazepines for only a very limited amount of time. In addition to addiction, including the danger of potentially severe withdrawal symptoms, long-term benzodiazepine use is associated with side effects such as psychomotor retardation, cognitive impairment, affective symptoms, paradoxical inhibition, and drug interactions [33]. Again, elderly patients are at an increased risk for the occurrence of these side effects [33–35]; however, the prescription of benzodiazepines to patients simultaneously increases with increasing age [32, 36]. CNS effects, such as cognitive impairment and sedation, as well as drug interactions play a significant role in elderly patients [33, 35, 37]. Of particular concern is the increased risk of falls in elderly patients receiving benzodiazepine treatment [38, 39], with potentially life-threatening consequences.

The medical departments with the highest amounts of off-label use were departments with a focus on the treatment of acute cases and emergencies (emergency unit, trauma surgery, intensive care, and neurology/stroke unit). Psychiatric drugs in these situations were mostly given for acute sedation with protection from the stress and anxiety of the situation or/and prophylaxis of delirium. In emergency situations, there is usually no time for a medical clinician to consult a psychiatrist about the choice of drugs; thus, acute treatment decisions need to be made by physicians based on their previous knowledge. We recommend the establishment of treatment standards in departments according to the respective national guidelines, since recommendations may differ according to the status of licenses for different drugs in different countries. In example, the guidelines for “Analgesia, sedation and management of delirium” published by an interdisciplinary consortium of 12 German medical societies, including the German society of Psychiatry and Psychotherapy, Psychosomatics and Neurology (DGPPN) [40], recommends the carefully monitored use of benzodiazepines for symptom-oriented treatment of agitation and anxiety as well as sedation (alternatives in intensive care settings: Propofol and Clonidine). Neuroleptics are recommended for the treatment of psychotic symptoms only, for example, in delirium and are not to be regularly used as sedatives. A pharmacological prophylaxis of delirium is recommended for at-risk patients only using low-dose Haloperidol (as licensed). However, these recommendations can only provide very general guidelines, since treatment of individual patients in a given situation often requires acknowledgement of diverse factors, and the establishment of one standard treatment for all patients is difficult. Therefore, a regular review of cases with the consultation-liaison psychiatrist could improve patient care in the time following an acute situation and could help to train physicians on treatment decisions in future cases.

A limitation of our study is the lack of information on the exact causes of a prescription being off-label, since these data were not recorded at the time of data collection. Although information on factors such as dose, length of treatment, patient’s age, and means of administration were available to the psychiatrists and included in their assessment of off-label use at the time of data collection, the distribution of causes for prescriptions being off-label cannot be retrospectively analyzed from our database. Future studies of off-label medication use should include the detailed recording of this information to obtain a more differentiated picture of common, potentially problematic prescribing situations in different drug categories.

## Conclusions

In summary, the off-label prescription of psychotropic drugs, particularly antipsychotics and benzodiazepines, is a very common phenomenon in German hospital wards of non-psychiatric specialties, at least in the investigated hospitals. Furthermore, most of these prescriptions are not supported by solid clinical and scientific knowledge and may thus constitute a potential risk for patients, particularly when patients are administered multiple off-label medications. Better training on psychiatric pharmacology for physicians from other specialties is necessary, as is a policy for the timely consultation of a specialized psychiatric colleague in hospitalized patients presenting with a psychiatric pathology.

## Abbreviations

UK: United Kingdom
EPS: extrapyramidal symptoms
